# Common-garden metabolomics reveals germplasm-associated metabolic diversity and candidate resources for functional food development in dried jujube (*Ziziphus jujuba* Mill.)

**DOI:** 10.3389/fpls.2026.1980416

**Published:** 2026-10-14

**Authors:** Shiyong Wen, Wenjia Leng, Haiyang Wang, Liguo Qiu, Wenjiao Li, Lan Wang, Xingang Li, Cuiping Wen

**Affiliations:** 1 College of Life Scinece, Dezhou University, Dezhou, Shandong, China; 2 Shandong Agricultural Engineering University, Zibo, Shandong, China; 3 Dezhou Academy of Agricultural Sciences, Dezhou, Shandong, China; 4 Dezhou Guixin Environmental Protection Technology, Dezhou, Shandong, China; 5 College of Forestry, Northwest Agriculture and Forestry University, Yangling, Shaanxi, China

**Keywords:** common cultivation environment, dried jujube, functional metabolites, metabolic profiling, nutritional quality

## Abstract

**Background:**

Dried jujube (*Ziziphus jujuba* Mill.) is valued for its nutritional and medicinal properties. However, metabolic assessments of jujube germplasm based on materials collected from different geographical regions are often confounded by environmental variation, making it difficult to distinguish germplasm-associated metabolic differences from environmental effects. This study aimed to characterize metabolic diversity among dried jujube germplasm resources under common-garden conditions and identify candidate resources with favorable nutritional and functional metabolite profiles.

**Methods:**

A total of 66 dried jujube germplasm resources originating from diverse regions of China were cultivated under a common environment and evaluated using LC-MS/MS-based broad-targeted metabolomics. Metabolic profiles were characterized by principal component analysis, uniform manifold approximation and projection, and hierarchical clustering. The accumulation patterns of major nutritional and functional metabolite classes were further compared for comprehensive evaluation of germplasm resources.

**Results:**

A total of 460 metabolites were annotated, mainly including organic acids, terpenoids, flavonoids, nucleotides and derivatives, and lipids. Multivariate analyses revealed distinct metabolic profiles among germplasm resources, whereas metabolic clustering did not fully correspond to geographical origin, indicating substantial germplasm-associated variation under common-garden conditions. Organic acids, flavonoids, phenolic acids, triterpenoids, and alkaloids showed relatively large variation among germplasm resources and contributed substantially to differences in metabolic and nutritional profiles. SHX-J-C, SHX-Y-X, SHX-Y-D, and LN-G-D showed favorable profiles of key nutritional and functional metabolites. Comprehensive evaluation further identified candidate germplasm resources with distinctive metabolic characteristics and potential for targeted utilization.

**Conclusions:**

Common-garden metabolomics revealed substantial metabolic diversity among dried jujube germplasm resources beyond geographical patterns, supporting an important contribution of germplasm background to metabolic variation. The identified candidate resources provide valuable materials for further quality evaluation, breeding, and targeted utilization in functional food development.

## Introduction

Jujube (*Ziziphus jujuba* Mill.) is an economically important fruit crop native to China and a valuable horticultural resource with applications in food, medicine, and functional product development (Huang et al., 2015; Liu et al., 2020). With a cultivation history of more than 4,000 years, jujube has become one of China’s most widely distributed and cultivated specialty fruit crops because of its broad ecological adaptability, strong stress tolerance, and rich nutritional composition (Huang et al., 2016). Growing consumer demand for natural functional foods has further increased interest in jujube fruits, which are rich in bioactive compounds, including soluble sugars, organic acids, vitamins, flavonoids, triterpenoids, phenolic acids, polysaccharides, and alkaloids (Rashwan et al., 2020). Jujube polysaccharides exhibit diverse structural characteristics and have been reported to possess antioxidant activities, highlighting their potential functional and food-related applications (Ji et al., 2020b; Ji et al., 2020a). These metabolites contribute to the nutritional and sensory properties of jujube and may also confer health-promoting effects, making the fruit an important resource for functional foods, bioactive compound extraction, and medicinal–food applications (Lu et al., 2021; Cai et al., 2024; Zhu et al., 2024). Based on their primary uses, jujube cultivars are generally classified as fresh or dried types. Compared with fresh jujube cultivars, dried types typically have higher dry matter content, better processing adaptability, and greater suitability for dried fruit production, making them important germplasm resources for the dried jujube industry and functional food applications (Pareek, 2013; Cai et al., 2024). Whereas fresh jujube is valued mainly for its appearance, texture, and flavor, dried jujube is primarily evaluated based on its nutritional composition, processing characteristics, and functional properties. These attributes are influenced by genetic background, metabolic composition, and the accumulation of bioactive compounds (Jia et al., 2024). However, current evaluations of jujube germplasm largely rely on conventional agronomic and physicochemical traits, such as single fruit weight and soluble solids content, which provide limited information on cultivar-specific metabolic diversity and functional metabolite accumulation (Shao et al., 2024). Therefore, systematic characterization of the metabolic profiles of dried jujube germplasm is essential for developing a comprehensive quality evaluation system, identifying elite cultivars with superior nutritional potential, and supporting the sustainable development of the dried jujube industry.

Fruit quality is a complex trait determined by interactions among genetic background, metabolic regulation, and environmental factors. Beyond conventional attributes such as fruit size and sugar–acid balance, quality depends on metabolic networks that shape nutritional composition and functional properties. Metabolomic studies have shown that fruit quality is closely associated with the coordinated accumulation of primary and secondary metabolites, whose profiles provide an integrated view of nutritional value, flavor, and potential health benefits (Fiehn, 2002; Zhang and Hao, 2020). Thus, metabolomics is increasingly used to elucidate the mechanisms of quality formation, identify superior germplasm, and support the development of functional foods (Li et al., 2022; Wang et al., 2023). During fruit maturation, primary metabolites, including sugars, organic acids, and amino acids, are central to carbon and nitrogen metabolism, energy supply, and flavor development and therefore strongly influence sweetness, acidity, and processing quality. Secondary metabolites, including flavonoids, triterpenoids, phenolic acids, alkaloids, lipids, and nucleotide derivatives, contribute to antioxidant, anti-inflammatory, and other health-promoting activities and are important indicators of nutritional and functional quality (Lu et al., 2017; Cai et al., 2024). In jujube fruits, flavonoids and pentacyclic triterpenoids are among the most extensively studied bioactive compounds. Flavonoids contribute to stress responses, reactive oxygen species scavenging, and antioxidant activity, whereas pentacyclic triterpenoids, such as ursolic, oleanolic, and corosolic acids, exhibit diverse biological activities, including anti-inflammatory, hypoglycemic, and cardioprotective effects (Lu et al., 2021; Wen et al., 2023). Additionally, other metabolites, including organic acids, lipids, alkaloids, and nucleotide derivatives, contribute to flavor, energy metabolism, signaling, and defense, collectively shaping jujube fruit quality (Miao et al., 2024; Guo et al., 2026). Therefore, comprehensive metabolomic profiling of dried jujube cultivars can provide deeper insights into the metabolic basis of quality than conventional physicochemical indicators and facilitate the identification of germplasm with superior nutritional and functional properties. Such analyses can also support comprehensive quality evaluation and the breeding of elite dried jujube cultivars with enhanced nutritional value and functional potential.

The metabolic composition and bioactive profiles of jujube are influenced by fruit development, genetic background, environmental conditions, and postharvest factors. Fruit development is particularly important because it involves extensive metabolic reprogramming. Integrated metabolomic and transcriptomic analyses of developing ‘Jinsixiaozao’ fruitsrevealed dynamic changes in flavonoids, phenolic acids, organic acids, and lipids, indicating coordinated regulation of multiple metabolic pathways during fruit maturation (Shen B. et al., 2022). Genetic background also contributes substantially to metabolic diversity among cultivars. Comparative metabolomic studies have identified marked genotype-dependent differences in phenolics, flavonoids, lipids, and organic acids. For example, untargeted metabolomic profiling of 11 jujube cultivars identified more than 1,300 metabolites and revealed distinct cultivar-specific metabolic signatures, demonstrating the potential of metabolomics for cultivar discrimination and quality evaluation (Yao et al., 2023). Similarly, integrated widely targeted metabolomics and network pharmacology identified over 1,500 metabolites across diverse jujube cultivars, with substantial variation in flavonoids, terpenoids, phenolic compounds, and other bioactive metabolites. These findings highlight the metabolic diversity and functional potential of jujube germplasm (Chen et al., 2026).

Environmental conditions also strongly influence the metabolic composition of jujube fruits. Geographic origin, climate, and ecological conditions can substantially affect the accumulation of nutritional and functional metabolites. A widely targeted metabolomic analysis of dried jujube from seven major production regions in China identified over 460 metabolites and revealed significant regional differences in flavonoids, triterpenoids, organic acids, amino acids, and other functional metabolites, enabling the identification of metabolite characteristics associated with superior production regions (Shi et al., 2022). Similarly, comprehensive metabolic profiling of fresh jujube from 11 geographical regions showed that climatic factors, ecological conditions, and cultivation regions significantly influenced fruit metabolic composition and nutritional quality, further emphasizing the role of environmental variation in jujube fruit quality formation (Wen et al., 2026). However, fruit metabolic profiles ultimately reflect the combined effects of genotype and environment, making it difficult to attribute metabolic differences among samples from different geographical locations to genetic variation alone. Common garden experiments have therefore emerged as an effective approach for disentangling genetic and environmental effects on quality-related metabolic traits. Studies in various fruit crops, including citrus, apple, and grape, have shown that cultivars grown under identical environmental conditions can still exhibit substantial differences in sugars, organic acids, polyphenols, carotenoids, volatile compounds, and other metabolite classes. These genotype-dependent metabolic differences are closely associated with fruit quality, sensory properties, and germplasm utilization potential (Kim et al., 2023; Gomez et al., 2024; Michailidis et al., 2024). Collectively, these findings demonstrate that common garden approaches can minimize environmental variation and provide a more reliable framework for identifying genetic contributions to metabolic diversity, thereby enabling more accurate evaluation of fruit germplasm resources and identification of elite cultivars.

Despite these advances, systematic metabolomic characterization of dried jujube germplasm remains limited. Previous studies have focused mainly on developmental stages, geographical origins, or processing conditions, whereas large-scale collections maintained under uniform cultivation conditions remain poorly characterized. Environmental differences in climate, soil, cultivation practices, and fruit maturity can confound the assessment of genetic contributions to metabolic variation. Moreover, studies have largely examined individual functional compounds or specific quality traits, with limited integration of diverse nutritional metabolites for comprehensive quality evaluation and candidate germplasm resources identification. Here, 66 dried jujube cultivars conserved in a single germplasm repository were analyzed by LC-MS/MS-based metabolomics to characterize cultivar-specific metabolic profiles and diversity under uniform cultivation conditions. Multivariate statistical analyses and nutritional quality assessments were used to identify key differential metabolites and candidate germplasm resources. Therefore, these findings provide insights into the genetic basis of metabolic variation in dried jujube and support germplasm improvement, quality breeding, and the development of functional jujube products.

## Materials and methods

### Plant materials and dried jujube sample preparation

A total of 66 dried-jujube cultivars were selected from the jujube germplasm collection (**Supplementary Table S1**). All cultivars were grown under identical conditions and standardized field management at the Jujube Germplasm Resource Nursery in Taigu, Shanxi Province, China. The nursery is located at 37°23′N, 112°32′E, at an elevation of approximately 830 m, with a mean annual temperature of approximately 10.6 °C. The site is located on the Loess Plateau, and the soil is predominantly sandy loam (pH approximately 7.5). The cultivars originated from 11 regions of China: Guangdong (GD), Gansu (GS), Guizhou (GZ), Henan (HN), Hunan (HUN), Shanxi (SX), Shaanxi (SHX), Shandong (SD), Liaoning (LN), Xinjiang (XJ), and Yunnan (YN) (**Supplementary Figure S1**). Their cultivation under a common-garden environment reduced environmental heterogeneity and provided a standardized basis for comparing germplasm-associated differences in metabolite accumulation.

Fruits were harvested at commercial maturity. For each cultivar, three independent trees were selected, and 20 uniform and undamaged fruits were randomly collected from each tree. The 20 fruits collected from each tree were pooled to constitute one biological replicate, resulting in three biological replicates per cultivar, with each biological replicate representing an independent tree. After seed removal, the edible tissues from each biological replicate were pooled, immediately frozen in liquid nitrogen, finely powdered, and stored at −80 °C until metabolite extraction. The samples were processed and stored on a fresh-weight basis, and the moisture content of the fruit tissues was not independently determined before freezing.

### Metabolite extraction

Approximately 100 mg of freeze-dried powder from each biological replicate was accurately weighed and extracted with 1.0 mL of chilled 80% (v/v) aqueous methanol. Samples were thoroughly vortex-mixed and subsequently subjected to static extraction at 4 °C for 12 h. The extracts were then centrifuged at 12,000 × g for 10 min at 4 °C. The resulting supernatants were carefully collected and filtered through 0.22 μm membrane filters into amber vials prior to LC-MS/MS analysis. Three biological replicates were analyzed for each variety, each representing an independent tree. Each biological replicate was analyzed by LC-MS/MS using a single injection. A pooled quality control (QC) sample was prepared by combining aliquots of the experimental samples and was injected after every 10 experimental samples throughout the analytical sequence to monitor the stability and reproducibility of the analytical platform. The relative standard deviation (RSD) of feature peak areas across the pooled QC injections was calculated as a measure of analytical reproducibility. Features with an RSD ≤30% across the pooled QC samples were considered to meet the predefined QC acceptance criterion and were retained for subsequent data processing and statistical analyses. Metabolites were subsequently annotated based on metabolite databases and/or MS/MS information.

### LC-MS/MS detection

Metabolite profiling was performed using an ultra-performance liquid chromatography coupled with tandem mass spectrometry system. Chromatographic separation was achieved on a Shim-pack UFLC system (Shimadzu, Japan) equipped with a Waters ACQUITY UPLC HSS T3 C18 column (1.8 μm, 2.1 × 100 mm) maintained at 40 °C. The mobile phase consisted of solvent A (water containing 0.04% acetic acid) and solvent B (acetonitrile containing 0.04% acetic acid). The gradient elution program was set as follows: 95% solvent A at 0 min, decreased linearly to 5% at 11.0 min, maintained until 12.0 min, and returned to 95% solvent A from 12.1 to 15.0 min for column equilibration. The flow rate was maintained at 0.4 mL/min, and the injection volume was 2 μL. Mass spectrometric detection was conducted using an Applied Biosystems 6500 QTRAP triple quadrupole-linear ion trap mass spectrometer (AB Sciex, Framingham, MA, USA) equipped with an electrospray ionization (ESI) source. The ion source temperature was set at 500 °C, with an ion spray voltage of 5500 V. Curtain gas pressure was maintained at 25 psi, and collision-induced dissociation (CID) was performed under high-energy conditions. Multiple reaction monitoring (MRM) transitions were optimized for each metabolite according to the declustering potential (DP) and collision energy (CE).

### Metabolite identification and annotation

Metabolites were annotated using a widely targeted LC-MS/MS metabolomics approach. The reference database was established using authentic standards and contained characteristic precursor ion and MS/MS fragmentation information. Metabolites detected in jujube samples were annotated by matching their precursor ion and MS/MS information against this authentic-standard-derived reference database. The 460 metabolites are therefore referred to as annotated metabolites throughout the manuscript. Detailed annotation information is provided in **Supplementary Table S3**.

### Principal component analysis (PCA)

In order to evaluate the differences in overall metabolic composition among different varieties of dried jujubes, PCA was conducted based on the normalized relative abundance data of all annotated metabolites. PCA converts multidimensional metabolic data into principal components through dimensionality reduction to visualize overall metabolic patterns and clustering relationships among samples.

### Uniform manifold approximation and projection (UMAP) analysis

UMAP was performed to further visualize nonlinear relationships among cultivars based on metabolomic profiles (Lim and Qiu, 2023). UMAP analysis was conducted using normalized metabolite abundance matrices, allowing visualization of potential metabolic similarities and differentiation patterns among different germplasm resources.

### Correlation analysis and metabolite–cultivar association analysis

To evaluate the similarity of metabolic composition among different varieties of dried jujubes, a correlation analysis was conducted based on the normalized relative abundance of all detected metabolites. The Pearson correlation coefficient was used to evaluate the metabolic correlation between different samples. The higher the correlation coefficient, the more similar the metabolic composition characteristics of the two varieties. In addition, a metabolite-variety association network is constructed based on the information of metabolite categories and their relative contents. Through the network relationship, the distribution patterns of characteristic metabolites in different dried jujube varieties are analyzed, as well as the contribution of major metabolic categories to the formation of variety differences. Correlation analysis and network visualization analysis were accomplished using the OmicStudio online analysis platform.

### Hierarchical clustering analysis (HCA)

HCA was conducted based on standardized metabolite abundance data. Euclidean distance was used to evaluate metabolic dissimilarity among samples, and clustering was performed using the hierarchical clustering algorithm. The resulting dendrogram was used to classify cultivars with similar metabolic profiles and reveal metabolic relationships among different germplasm resources.

### KEGG pathway enrichment analysis

To further elucidate the potential biological functions involved in the metabolites of dried jujubes, KEGG database annotation and pathway enrichment analysis were conducted on the identified metabolites. According to the KEGG numbers corresponding to the metabolites, the detected metabolites are mapped to the relevant metabolic pathways, and the quantity and significance level of enriched metabolites in different pathways are statistically analyzed. The KEGG enrichment results are presented by drawing advanced bubble charts based on the *p*-values.

### Integrated metabolite profile evaluation

Five major nutritional and functional metabolite classes, including flavonoids, triterpenoids, organic acids, nucleotides and their derivatives, and lipids, were selected for integrated evaluation. For each class, cultivars were ranked according to cumulative relative metabolite abundance, with rank 1 representing the highest abundance. Equal weights were assigned to the five classes, and ranks were converted to scores using P_ij_=67-R_ij_. The integrated metabolite profile score for cultivar (i) was calculated as:

$S_{\text{i}}=\sum_{j=1}^{5}\left(67-R_{ij}\right),$where R_ij_ is the rank of cultivar (i) for class (j). Higher scores indicate relatively higher cumulative abundance across the selected classes. The score was used for comparative profiling rather than as an absolute measure of nutritional value. Because the selection of metabolite classes and equal weighting involved predefined methodological assumptions, the resulting score was used only as a comparative metabolite profiling index rather than as an objective measure of nutritional value.

### Statistical analysis and metabolomics data processing

Metabolite peak-area data were normalized to total ion intensity, log2-transformed, and Pareto-scaled. Features with excessive missing values were excluded, and remaining missing values were imputed as one-half of the minimum detected value. Differential metabolites were identified using combined multivariate and univariate analyses. P-values were adjusted using the Benjamini–Hochberg method, and metabolites with VIP ≥1, |log2FC| ≥1, and FDR<0.05 were considered significant. Both raw and FDR-adjusted P-values were reported. Data processing and visualization were performed using TBtools, OriginPro 2021, and OmicStudio (accessed 15 March 2026).

## Results

### Metabolic diversity and genetic specificity of dried jujube germplasm under a common garden environment

To systematically assess the metabolic diversity of dried jujube germplasm, 66 cultivars from 11 major production regions in China, including GD, GS, GZ, HN, HUN, SHX, SX, SD, LN, XJ, and YN, were analyzed using widely targeted metabolomics. Although the cultivars originated from geographically distinct regions, all were grown under identical environmental and management conditions in the same germplasm repository before sampling. Thus, the observed metabolic differences primarily reflect genotype-associated variation rather than environmental effects.

LC-MS/MS-based widely targeted metabolomics detected and annotated 460 metabolites across the 66 jujube germplasm resources. Metabolite assignments were based on matching precursor ion and characteristic MS/MS fragmentation information against an authentic-standard-derived reference database. The annotated metabolites included organic acids, flavonoids, phenolic acids, triterpenoids, alkaloids, lipids, amino acids and their derivatives, nucleotides and their derivatives, carbohydrates, and vitamins (**Supplementary Table S3**). These metabolites represented diverse metabolic classes and showed substantial variation in abundance among the different germplasm resources. Principal component analysis (PCA) based on the 460 metabolites clearly separated the metabolic profiles of the 66 cultivars (**Figure 1a**), with PC1 and PC2 explaining 40.67% and 32.23% of the total variation, respectively. The cultivars did not cluster according to geographical origin but were widely distributed in the PCA plot, indicating that distinct metabolic phenotypes were maintained under uniform cultivation conditions. This pattern suggests that the observed metabolic differences were associated with germplasm-specific variation under the common-garden conditions, although residual environmental effects cannot be completely excluded. To further characterize the intrinsic structure of the high-dimensional metabolomic dataset, UMAP was performed. UMAP further resolved the 66 cultivars into several relatively distinct clusters and provided greater local separation than PCA (**Figure 1b**), highlighting the complex metabolic structure and substantial heterogeneity of the germplasm collection.

**Figure 1:**
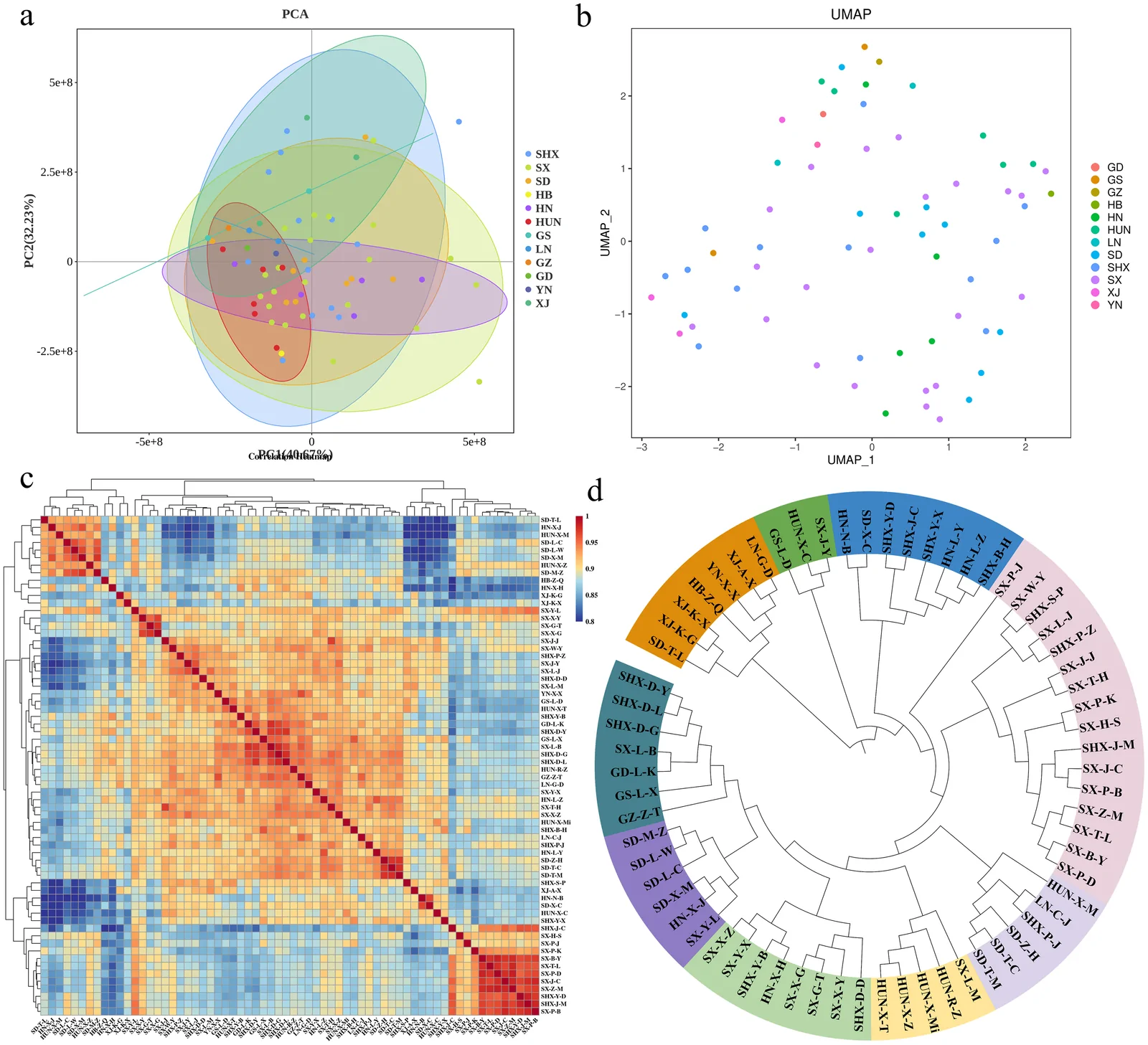
Metabolic diversity and genetic relationships among 66 dried jujube germplasm resources. **(a)** PCA based on the relative abundance of 460 annotated metabolites. **(b)** UMAP showing the nonlinear distribution of metabolomic profiles. **(c)** Pearson correlation heatmap illustrating metabolic similarity among cultivars. **(d)** HCA based on normalized metabolite abundance. Cultivar abbreviations correspond to their codes listed in **Supplementary Table S1**.

Pairwise correlation analysis revealed considerable variation in metabolite accumulation patterns among cultivars (**Figure 1c**). Most cultivars showed moderate to high metabolic similarity, whereas several pairs exhibited low correlation coefficients, indicating marked metabolic divergence. Hierarchical clustering based on all detected metabolites further revealed the metabolic relationships among cultivars (**Figure 1d**). Four Xinjiang cultivars (XJ-K-G, XJ-K-X, XJ-X-Q, and XJ-X-T) clustered with the Shandong cultivar SD-T-L, indicating highly similar metabolic profiles. Similarly, GS-L-D, LN-G-D, XJ-X-F, and SX-X-J formed a distinct cluster despite their different geographical origins. Another major cluster comprised SHX-D-Y, SHX-D-L, and SHX-D-G, together with GS-L-X and GZ-L-Z, which shared similar metabolic profiles. In contrast, HN-X-J, SX-Y-L, SX-G-Q, SX-G-T, and SX-X-Z formed a distinct branch with markedly different metabolic profiles. Interestingly, cultivars from the same region were often distributed across different branches, whereas cultivars from different regions, such as SHX-Y-D, HN-X-H, SX-X-S, HN-X-T, HN-X-Z, and HN-X-M, clustered closely because of their similar metabolic profiles. These clustering patterns were consistent with the PCA and UMAP results, demonstrating that dried jujube cultivars possess distinct germplasm-associated metabolic variation under common-garden conditions. Overall, the observed metabolic variation was associated with germplasm-specific differences under the common-garden conditions, although residual environmental effects cannot be completely excluded.

### Functional characterization of metabolic pathways and metabolite association networks among dried jujube cultivars

To further characterize the metabolic basis of the genotype-dependent variation identified above, all 460 annotated metabolites were subjected to Kyoto Encyclopedia of Genes and Genomes (KEGG) pathway analysis (**Figure 2a**; **Supplementary Table S2**). The metabolites were mainly associated with Metabolic pathways and Biosynthesis of secondary metabolites, followed by ABC transporters, Phenylpropanoid biosynthesis, Flavonoid biosynthesis, Lipid metabolism, and Amino acid metabolism. These results indicate that metabolic differences among dried jujube cultivars involve both primary metabolism and secondary metabolite biosynthesis. Pathways related to the biosynthesis of bioactive compounds, particularly flavonoids, phenolic acids, and triterpenoids, were prominently enriched, suggesting that these metabolite groups contribute substantially to cultivar-specific metabolic diversity. To further characterize the relationships between cultivars and metabolite classes, we constructed a cultivar–metabolite association network (**Figure 2b**). The network showed extensive associations between individual cultivars and diverse metabolite classes. Organic acids, flavonoids, phenolic acids, triterpenoids, alkaloids, and carbohydrates were associated with multiple cultivars, whereas each cultivar was connected to several metabolite classes. Thus, the metabolic profiles of dried jujube cultivars appear to reflect the coordinated accumulation of diverse metabolites rather than the predominance of a single metabolite class. This broad metabolic connectivity highlights the complex regulation of metabolism and the substantial biochemical diversity within the jujube germplasm collection.

**Figure 2:**
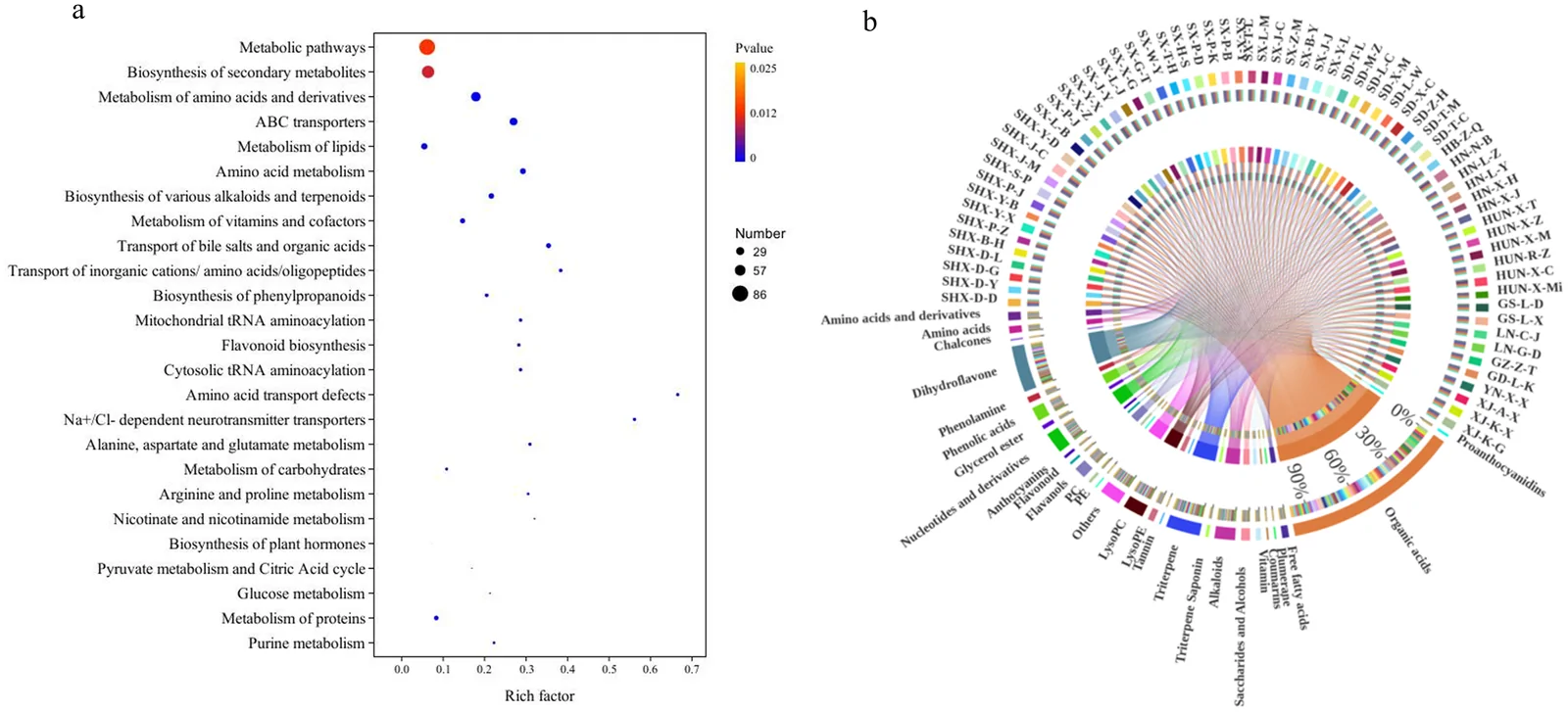
Functional characterization of identified metabolites and cultivar–metabolite associations. **(a)** KEGG pathway classification of the 460 identified metabolites. Bubble size represents the number of annotated metabolites, bubble color indicates enrichment significance, and the Rich factor was calculated as the ratio of the number of target metabolites annotated to a given KEGG pathway to the total number of metabolites annotated to that pathway. **(b)** Cultivar–metabolite association network showing the relationships between dried jujube cultivars and major metabolite classes.

### Accumulation patterns of functional metabolites reveal metabolic diversity and nutritional characteristics among dried jujube cultivars

Based on the extensive associations identified by pathway enrichment and network analyses, the accumulation patterns of different metabolite classes were further compared across the 66 dried jujube cultivars (**Figure 3**). Clear cultivar-dependent differences were observed among metabolite classes. Organic acids, flavonoids, phenolic acids, triterpenoids, and alkaloids varied substantially in abundance, whereas primary metabolites, including amino acids and their derivatives and nucleotides and their derivatives, were comparatively stable. These results indicate that individual dried jujube cultivars possess distinct metabolic profiles, particularly in the relative abundance of bioactive secondary metabolites.

**Figure 3:**
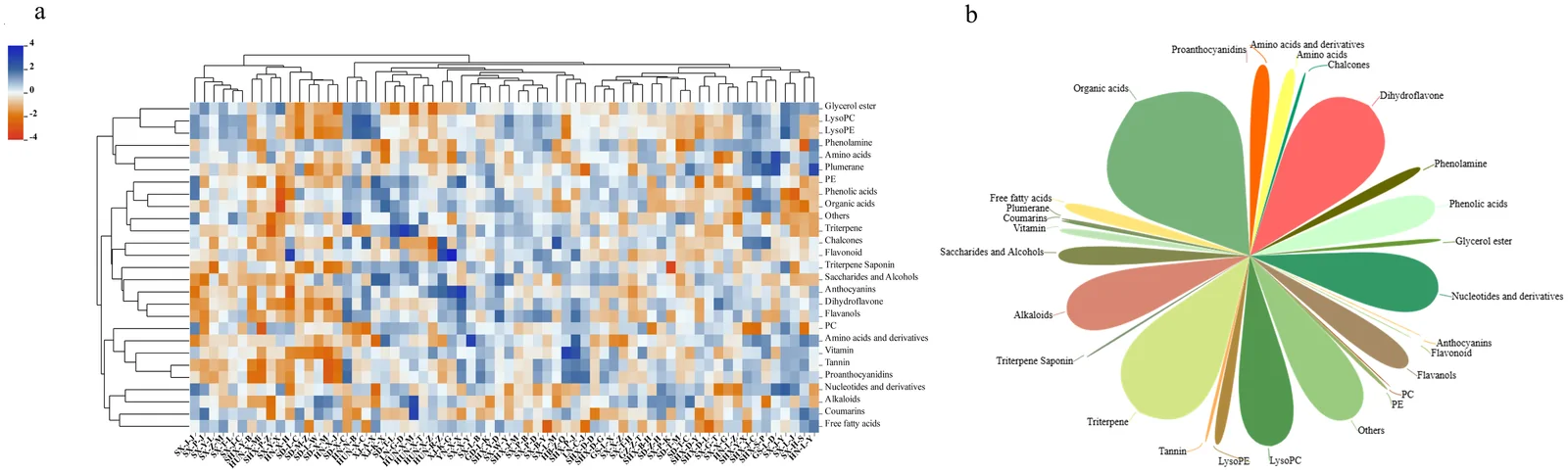
Accumulation patterns and composition of major metabolite classes among 66 dried jujube cultivars. **(a)** Heatmap showing the relative abundance of representative metabolites from different metabolite classes across cultivars. **(b)** Relative contribution of major metabolite categories to the total metabolite composition in each cultivar.

Among the major functional metabolite groups, organic acids showed pronounced cultivar-specific variation. Citric acid, malic acid, quinic acid, and related metabolites accumulated at relatively high levels in GS-L-X, SHX-Y-X, and SHX-J-C, but at much lower levels in HN-X-H, SHX-S-P, and SX-Z-M. Flavonoids also showed strong genotype-dependent variation, with YN-X-X, XJ-K-X, and SHX-P-J exhibiting consistently higher levels than most other cultivars, whereas several cultivars from Henan and Shanxi showed comparatively lower levels. Triterpenoids, which are characteristic bioactive metabolites of *Ziziphus* species, likewise varied substantially among cultivars (**Figure 3a**). Higher triterpenoid levels were observed in SHX-Y-X, LN-G-D, and SX-J-Y, whereas several other cultivars showed relatively low levels. Overall, these results demonstrate substantial cultivar-dependent variation in the accumulation of key functional metabolites, resulting in distinct metabolic characteristics among germplasm resources. Several cultivars showed concurrent enrichment of multiple functional metabolite classes. For example, SHX-Y-X accumulated high levels of organic acids, phenolic acids, and triterpenoids; YN-X-X was characterized by particularly high flavonoid levels; and LN-G-D showed a pronounced advantage in triterpenoid accumulation. In contrast, HN-X-H, SX-Z-M, and SHX-S-P consistently exhibited relatively low levels across several nutritionally relevant metabolite classes.

To further assess the contribution of different metabolite classes to the nutritional characteristics of dried jujube cultivars, metabolite classes were ranked by cumulative abundance (**Figure 3b**). Organic acids, terpenoids, flavonoids, nucleotides and their derivatives, and lipids were the predominant classes. Organic acids and terpenoids accounted for the largest proportions, whereas flavonoids and other bioactive secondary metabolites also made substantial contributions. The coordinated relative abundance patterns of these metabolite classes may provide useful indicators of the metabolic and functional profiles of dried jujube germplasm and support comparative germplasm evaluation.

### Overall metabolite accumulation and compositional profiles among dried jujube cultivars

To comprehensively characterize the metabolic profiles of dried jujube germplasm, the total relative abundance of the 460 annotated metabolites and the distribution of major metabolite classes were compared among the 66 cultivars (**Figure 4**). The summed relative abundance of the annotated metabolites varied considerably among cultivars, indicating differences in their overall metabolomic profiles. SHX-J-C, SHX-Y-X, SX-J-Y, XJ-K-X, HN-N-B, and SD-T-L showed relatively high summed metabolite abundance, whereas SX-Y-X, SHX-Y-B, and HN-X-H showed comparatively lower values (**Figure 4a**). Several cultivars with high total metabolite abundance also accumulated relatively high levels of functional metabolites, including flavonoids, phenolic acids, and triterpenoids, consistent with the findings described above. This pattern suggests that greater overall metabolite accumulation is associated with enrichment of nutritionally important bioactive compounds, highlighting these cultivars as potential germplasm resources for functional food development.

**Figure 4:**
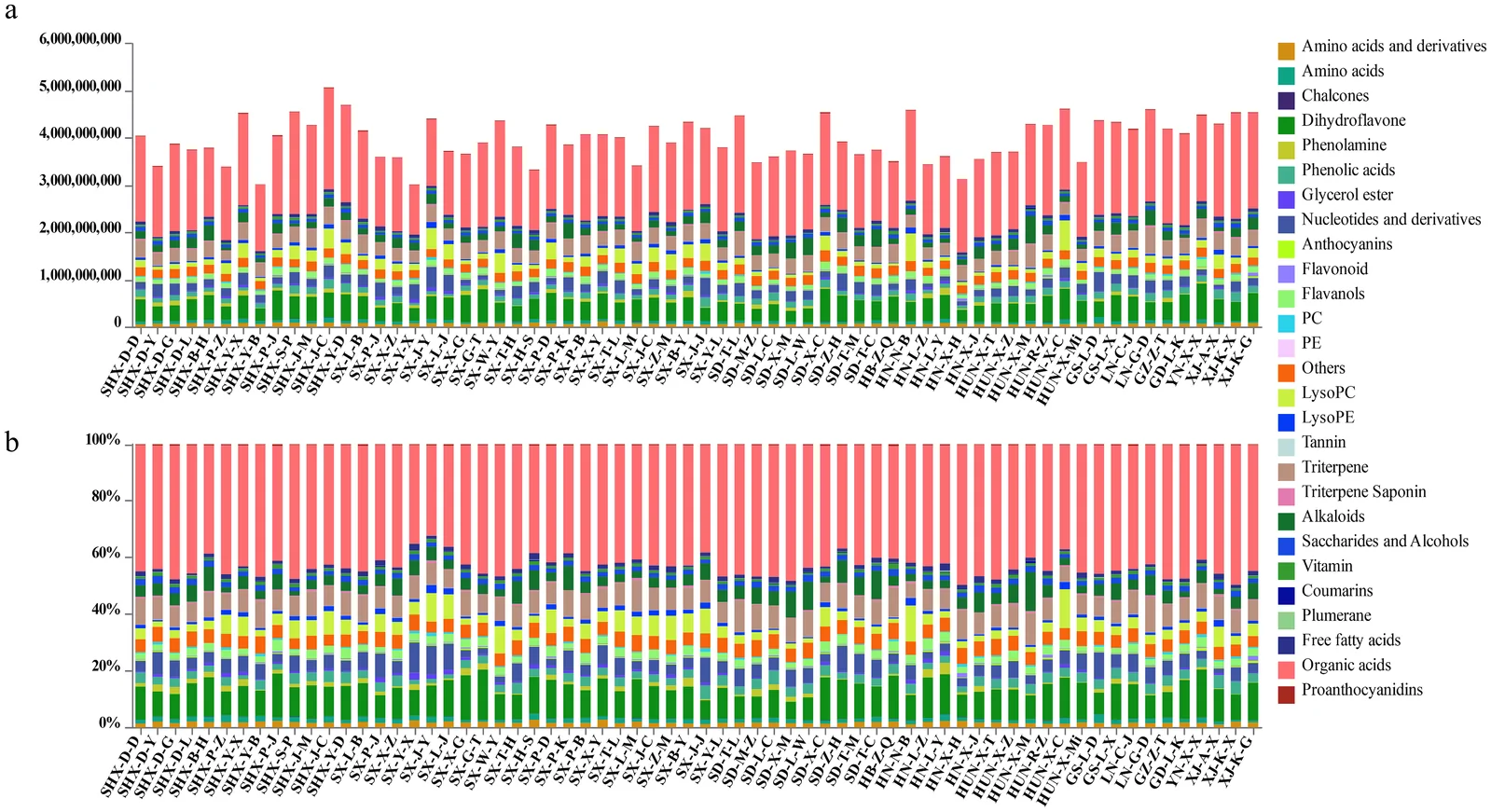
Overall metabolite abundance and metabolite composition of dried jujube cultivars. **(a)** Total relative abundance of metabolites across different metabolite classes in different jujube germplasms. **(b)** Relative proportion of different metabolite classes in each jujube germplasm.

The relative proportions of major metabolite classes were further compared among cultivars (**Figure 4b**). Although their overall metabolic profiles were broadly similar, the proportions of individual metabolite classes varied considerably. Organic acids were the predominant class in all cultivars and accounted for > 40% of total metabolite abundance in most samples. Flavonoids, terpenoids, phenolic acids, nucleotides and their derivatives, and lipids also contributed substantially, with some cultivars showing relatively high proportions of bioactive secondary metabolites. These differences indicate considerable variation in the nutritional composition of dried jujube germplasm. Integration of overall relative metabolite abundance and metabolite composition showed that SHX-Y-X, YN-X-X, LN-G-D, and SX-J-Y exhibited relatively high overall metabolite abundance together with elevated relative abundance of selected functional metabolite classes, particularly flavonoids, phenolic acids, and triterpenoids. These metabolic profiles may be of interest for further evaluation of their functional and utilization potential. Conversely, cultivars with lower overall relative metabolite abundance and lower proportions of selected functional metabolites showed distinct metabolic profiles that may warrant further characterization.

### Identification of candidate dried jujube germplasm based on integrated evaluation of key nutritional metabolites

To compare the nutritional and functional characteristics of dried jujube germplasm, radar plots were used to visualize the accumulation patterns of major metabolite classes across the investigated cultivars (**Figure 5**). The cultivars showed distinct metabolic profiles, particularly in organic acids, triterpenoids, flavonoids, nucleotides and their derivatives, lipids, and alkaloids, highlighting substantial diversity in the nutritional composition of the germplasm collection. SHX-Y-X, SHX-J-C, and SX-J-Y exhibited relatively high accumulation across multiple metabolite classes, indicating comparatively broad metabolic advantages. SHX-Y-X showed pronounced accumulation of organic acids, flavonoids, and triterpenoids, suggesting strong potential for bioactive metabolite enrichment. SHX-J-C maintained relatively high levels across most major nutritional metabolite classes, indicating a favorable overall metabolite profile, whereas SX-J-Y showed pronounced accumulation of several nutrition-related metabolites. In contrast, some cultivars exhibited consistently low accumulation across multiple metabolite classes, suggesting relatively limited nutritional and functional metabolite diversity.

**Figure 5:**
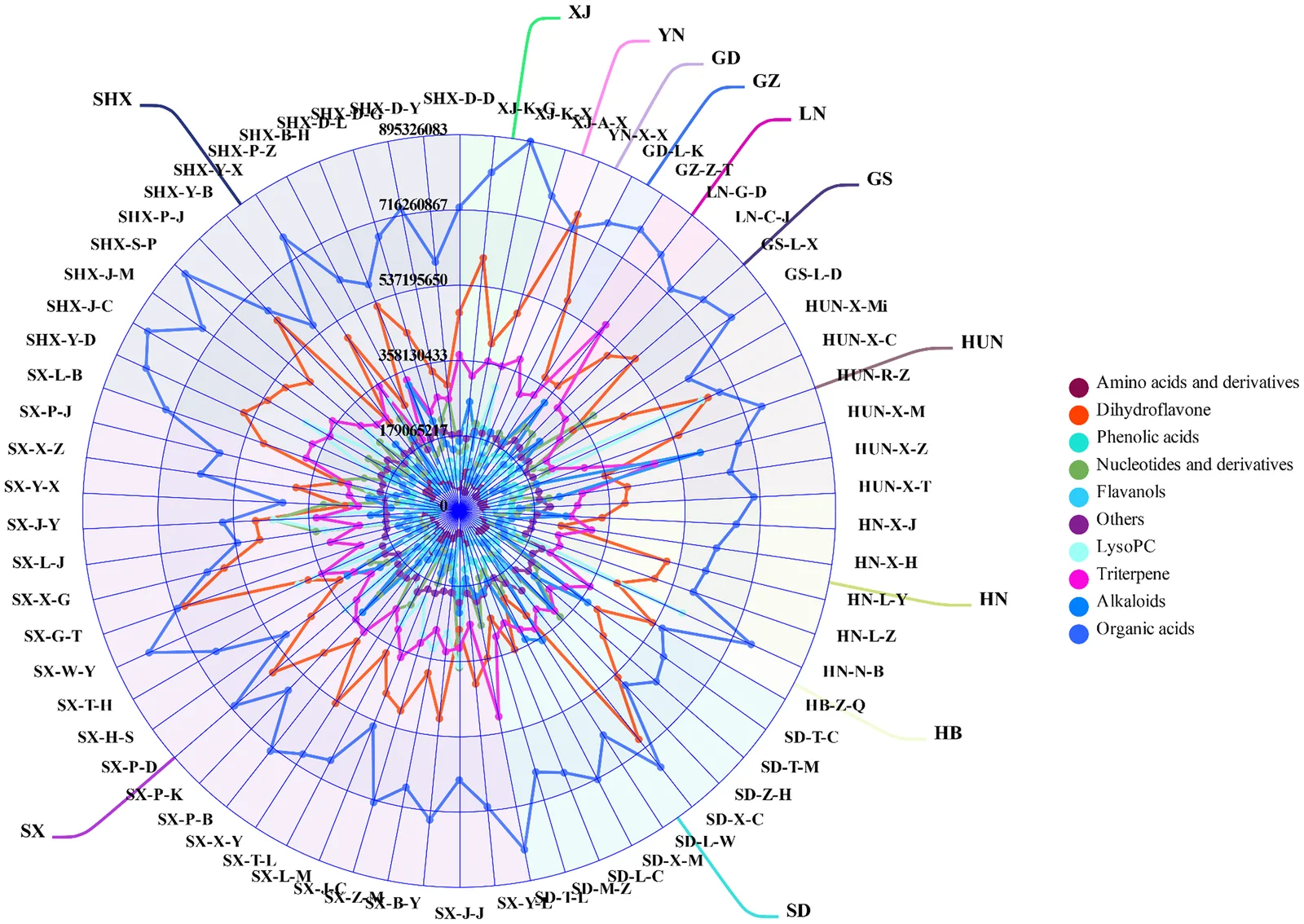
Comprehensive comparison of major nutritional metabolite categories among dried jujube cultivars.

To further characterize the metabolic basis of these cultivar-specific characteristics, representative nutritional metabolites were examined (**Figure 6**). The metabolic advantages of elite cultivars reflected the coordinated accumulation of multiple metabolite classes rather than enrichment of a single class. Organic acids, which constituted one of the major metabolite classes in dried jujube fruits, accumulated at relatively high levels in SHX-Y-X, LN-G-D, and SX-J-Y, potentially contributing to flavor and processing quality. Functional secondary metabolites showed greater cultivar specificity. YN-X-X, SHX-Y-X, and SX-J-Y accumulated relatively high levels of flavonoids, whereas LN-G-D, SHX-Y-X, and SHX-J-C showed elevated triterpenoid levels. Because flavonoids and triterpenoids are major bioactive compound classes in jujube fruits, these cultivars may represent promising germplasm resources for developing value-added functional products. Several cultivars also showed distinctive advantages in specific nutritional components. HN-L-Y and SHX-Y-D accumulated relatively high lipid levels; SX-J-Y was enriched in nucleotides and their derivatives; SX-X-Y showed relatively high levels of amino acids and their derivatives; and SHX-P-J accumulated more vitamins. XJ-A-X and XJ-K-X exhibited relatively high carbohydrate and phenolic acid levels, respectively, suggesting potential advantages in sensory attributes and functional properties (**Supplementary Figure S2**).

**Figure 6:**
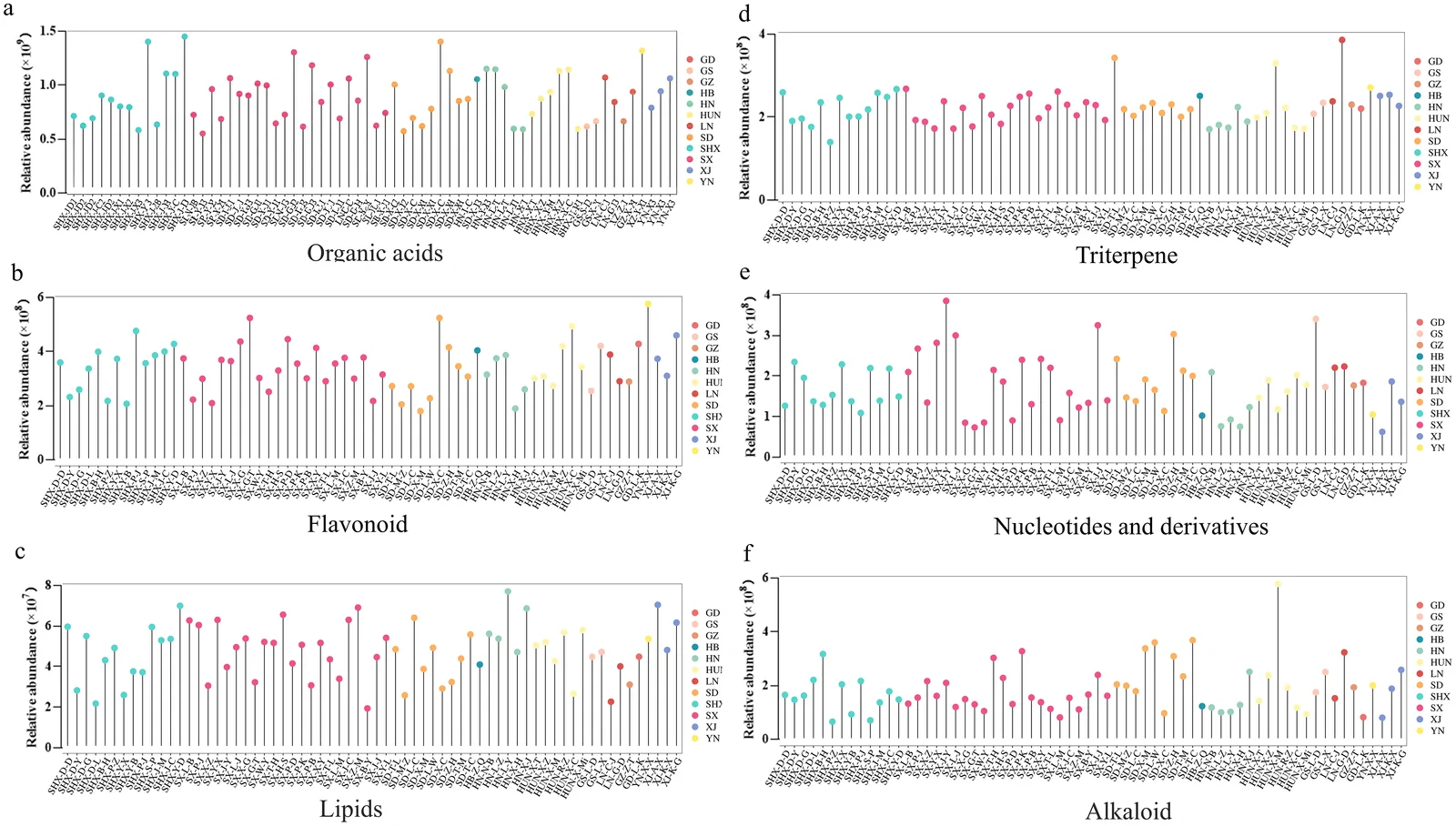
Comparison of representative nutritional metabolite categories among dried jujube cultivars. Relative abundance of representative metabolite categories, including **(a)** Organic acids, **(b)** Flavonoids, **(c)** Lipids, **(d)** Triterpene, **(e)** Nucleotides and their derivatives, **(f)** Alkaloid.

To further characterize the metabolite profiles associated with functional and nutrition-related metabolite classes, we established an integrated evaluation framework based on five major metabolite classes: flavonoids, triterpenoids, organic acids, nucleotides and their derivatives, and lipids. Cultivars were ranked according to the relative abundance profiles of these metabolite classes, and an integrated metabolite profile score was calculated for all 66 cultivars (**Supplementary Table S4**). This score reflects differences in the relative abundance and composition of the selected metabolite classes and should not be interpreted as a direct measure of absolute nutrient concentration or nutritional value. The index was intended to facilitate comparative profiling among cultivars, rather than to provide an objective nutritional ranking. The results revealed substantial variation in the integrated metabolite profiles among cultivars. SHX-J-C (279), SHX-Y-D (260), SHX-Y-X (256), LN-C-J, SHX-S-P, and SD-T-L showed relatively high integrated metabolite profile scores. These profiles reflected the combined relative abundance of multiple metabolite classes rather than a single metabolite class. SHX-J-C showed relatively high abundance across flavonoids, triterpenoids, organic acids, and nucleotide-related metabolites, whereas SHX-Y-X was characterized by relatively high abundance of organic acids, flavonoids, and triterpenoids. LN-G-D showed relatively high abundance of triterpenoids and organic acids. These findings were consistent with the radar plot and key metabolite analyses, further highlighting the distinct relative abundance profiles and potential functional relevance of these cultivars.

Overall, the dried jujube germplasm showed substantial variation in metabolite composition and relative abundance profiles. SHX-J-C, SHX-Y-X, SHX-Y-D, and LN-G-D exhibited relatively high overall metabolite abundance and coordinated relative abundance patterns across multiple bioactive metabolite classes. These characteristics highlight their potential as valuable germplasm resources for further evaluation and utilization in functional food development.

## Discussion

Fruit quality is determined by the complex interaction between genetic background and environmental conditions, making it difficult to accurately assess the intrinsic quality potential of germplasm resources. Previous studies of jujube and other fruit crops have often compared materials collected from different production regions, where differences in climate, soil properties, and cultivation practices can confound the effects of genotype and environment on metabolic variation. Evaluating diverse germplasms under uniform cultivation conditions therefore provides a useful approach for reducing environmental heterogeneity and comparing germplasm-associated metabolic variation. Metabolomics has emerged as a powerful tool for germplasm characterization because it enables comprehensive profiling evaluation of quality-related metabolites. For example, substantial differences in fatty acids, amino acids, and mineral nutrients among avocado germplasms have demonstrated the utility of metabolite profiling for distinguishing genetic resources with different nutritional properties (Yang et al., 2024). Similarly, pronounced differences in flavor-related metabolites among wild strawberry accessions grown under comparable environmental conditions indicate that stable metabolic phenotypes are largely determined by genetic background (Du et al., 2024). In the present study, 66 dried jujube germplasms originating from diverse ecological regions were cultivated under common-garden conditions to minimize environmental variation and enable systematic comparison of their metabolic profiles. PCA, UMAP, and hierarchical clustering consistently showed that the germplasms did not cluster according to their geographic origins. Instead, cultivars from geographically distant regions sometimes exhibited highly similar metabolic profiles, whereas accessions from the same region could be metabolically distinct. These patterns indicate that metabolic profiles differed substantially among germplasms even when the materials were evaluated under common-garden conditions. The lack of a clear correspondence between metabolic clustering and geographical origin is consistent with a contribution of germplasm-associated variation to metabolic diversity, although residual environmental effects cannot be completely excluded. Similar findings have been reported in tomato and other horticultural crops, in which genetically distinct cultivars maintained characteristic metabolic signatures under uniform cultivation conditions, further supporting the major contribution of genetic factors to fruit quality (Mun et al., 2021; Li et al., 2022). Therefore, integrating common-garden cultivation with metabolomic profiling provides a useful approach for characterizing germplasm-associated metabolic differences among dried jujube germplasms.

Conventional fruit quality assessment has typically focused on a limited set of indicators, such as soluble solids, the sugar-to-acid ratio, vitamin C, and total phenolic content. Although these parameters provide useful information, they capture only individual aspects of fruit quality and cannot fully represent its nutritional and functional properties. Increasing evidence indicates that fruit quality is determined by coordinated changes across multiple metabolic pathways, involving both primary and secondary metabolites, rather than by variation in a single compound or pathway (Hernández et al., 2016; Martinez-Rivas and Fernie, 2024; Singh et al., 2024). Our results support this multidimensional view of dried jujube quality. We annotated 460 metabolites across the 66 germplasms, spanning diverse biochemical classes, including organic acids, flavonoids, phenolic acids, triterpenoids, alkaloids, lipids, amino acids, and nucleotides. This broad metabolic diversity provides a comprehensive view of the metabolic characteristics associated with dried jujube fruit quality. Metabolic variation among germplasms was particularly associated with phenylpropanoid metabolism, flavonoid biosynthesis, lipid metabolism, and several primary metabolic pathways. These findings suggest that metabolic differences among dried jujube germplasms involve coordinated changes across interconnected metabolic pathways rather than variation in a single metabolic route. Notably, differences in the relative abundance of flavonoids, phenolic acids, and triterpenoids may be associated with variation in the functional metabolite profiles of dried jujube germplasms. These metabolite classes therefore provide useful indicators for comparative evaluation of functional potential.

Similar patterns of coordinated metabolic regulation have also been reported in other fruit crops. For example, systematic changes in sugars, organic acids, lipids, and secondary metabolites during peach processing and storage suggest that maintaining fruit quality depends on the coordinated regulation of multiple metabolic pathways (Wu et al., 2024). In apple, fruit quality preservation has likewise been associated with the coordinated regulation of organic acid metabolism and broader metabolic networks (Wang et al., 2024). Given that dried jujube is both a medicinal and edible fruit rich in diverse bioactive compounds, evaluating its quality based on only a few conventional physicochemical traits is unlikely to capture its full nutritional and functional value. A comprehensive analysis of multiple metabolite classes can therefore provide a more biologically relevant basis for evaluating quality and identifying germplasm with superior nutritional and functional potential.

As a medicinal and edible fruit rich in diverse bioactive compounds, jujube quality is determined not only by primary metabolites, such as sugars and organic acids, that contribute to taste and flavor, but also by bioactive secondary metabolites, including flavonoids, phenolic acids, and triterpenoids. Organic acids are important for determining fruit acidity, maintaining the sugar–acid balance, and regulating carbon metabolism during fruit development and ripening. In contrast, flavonoids and triterpenoids are major functional constituents because of their antioxidant activities and potential health-promoting effects, making them important indicators of functional fruit quality (Saito and Matsuda, 2010; Etienne et al., 2013; Fernie and Tohge, 2017; Shen N. et al., 2022; Zhao et al., 2025). Previous studies have shown that the nutritional and medicinal value of jujube is closely associated with its abundant organic acids, flavonoids, and triterpenoids. For example, metabolomic analyses of fresh jujube have revealed substantial differences in the accumulation of diverse nutritional metabolites among germplasms from different production regions, enabling the identification of accessions with superior functional potential (Wen et al., 2026). Similarly, pronounced differences in nutritional metabolite accumulation have been observed among dried jujube cultivars (Shi et al., 2022). However, these studies were mainly conducted using materials grown under natural environmental conditions. Thus, the observed metabolic differences reflect the combined effects of genetic background and environmental conditions, making it difficult to disentangle their respective contributions.

In the present study, 66 dried jujube germplasms were evaluated under common-garden conditions to minimize environmental variation and facilitate comparison of their intrinsic metabolic characteristics. Significant differences in major functional metabolite classes, including organic acids, flavonoids, and triterpenoids, were consistently observed among germplasms under common-garden conditions, suggesting that germplasm-associated variation contributes substantially to differences in dried jujube functional metabolite profiles. Notably, the candidate germplasm resources identified in this study did not show exceptionally high accumulation of any single metabolite class. Rather, they exhibited consistently favorable profiles across multiple functional metabolite categories, suggesting that the nutritional and functional quality of dried jujube is determined by the coordinated accumulation of diverse metabolites rather than by the enrichment of individual compounds (Zhang et al., 2022). These findings advance our understanding of jujube quality formation by demonstrating that superior quality is associated with a stable genetic basis in addition to environmental adaptation. They also provide a theoretical basis for the identification, conservation, and utilization of elite dried jujube germplasm.

An important goal of fruit germplasm evaluation is to characterize metabolic variation among diverse resources and identify candidate germplasm resources for further evaluation and utilization. Conventional fruit quality assessment generally relies on a limited set of parameters, such as soluble solids content, sugar–acid ratio, vitamin C, and total phenolic content. Although these indicators provide useful information on specific quality attributes, they cannot comprehensively capture the nutritional composition and functional value of dried jujube. Increasingly, integrated evaluations based on multiple metabolic traits have been recognized as an effective approach for elucidating fruit quality formation and identifying superior germplasm resources (Li et al., 2022; Sun et al., 2025). We established an integrated metabolite profiling framework based on the metabolic profiles of 66 dried jujube germplasms, covering major nutritional and functional metabolite classes, including organic acids, flavonoids, triterpenoids, phenolic acids, and amino acids. Significant differences were observed among germplasms in overall metabolite abundance and functional metabolite composition, with SHX-J-C, SHX-Y-X, SHX-Y-D, and LN-G-D showing relatively high integrated metabolite profile scores. Notably, these candidate germplasm resources were not characterized by excessive accumulation of a single metabolite class but instead showed favorable profiles across multiple functional metabolic categories. This finding suggests that the superior quality potential of dried jujube is likely determined by the coordinated contributions of multiple metabolic modules and that integrated metabolic profiles provide a more comprehensive assessment of germplasm value than individual quality indicators (Zhang et al., 2023; Zhou et al., 2023). The candidate germplasm resources identified in this study also differed from those reported in previous evaluations based on dried jujube samples collected from different production regions. Among germplasms from different regions, HN-L-L, XJ-H-Hm, and XJ-H-Hp were ranked highest based on comprehensive nutritional and metabolic evaluation (Shi et al., 2022). However, because these materials originated from distinct ecological environments, their metabolic characteristics likely reflected the combined effects of genetic background and environmental factors, including light, temperature, and precipitation. Thus, evaluations based on regional samples generally represent the integrated effects of genotype–environment interactions. In contrast, the germplasms in the present study were cultivated under identical common-garden conditions, thereby minimizing environmental variation. Therefore, the distinctive metabolic profiles observed for SHX-J-C, SHX-Y-X, SHX-Y-D, and LN-G-D may partly reflect germplasm-associated characteristics under the common-garden conditions. This may explain why some germplasms that perform exceptionally well under specific production environments do not necessarily maintain the highest rankings under uniform cultivation conditions, whereas accessions from regions without apparent ecological advantages may exhibit strong intrinsic quality potential. These findings should be interpreted with caution, as the evaluation was based on relative metabolite abundance profiles. The identified germplasm resources therefore require further targeted quantitative and phenotypic validation.

Similar findings have been reported in other fruit crops, where stable metabolic phenotypes obtained under common environmental conditions improved the reliability of core germplasm identification by minimizing environmental effects (Cao et al., 2022). Therefore, the integrated metabolic evaluation framework developed in this study provides a systematic approach for assessing dried jujube quality and identifying candidate germplasm resources. It also offers valuable resources for functional food development and germplasm improvement. Future integration of genome-wide association studies, transcriptomic analyses, and metabolic regulatory network analyses will help elucidate the genetic mechanisms underlying key nutritional quality traits and facilitate a shift from conventional phenotypic selection toward more precise evaluation and targeted improvement of dried jujube germplasm.

Several limitations should be considered when interpreting the present findings. The metabolomic evaluation was based on relative metabolite abundance, and the identified candidate germplasm resources have not yet been independently validated by targeted quantitative or phenotypic analyses. Therefore, the observed metabolic differences should not be interpreted as direct evidence of superior nutritional or functional performance. Future studies integrating targeted metabolite quantification with nutritional and phenotypic evaluations will help further validate the utilization potential of these germplasm resources.

## Conclusion

This study revealed extensive metabolic diversity among 66 dried jujube germplasm resources cultivated under common environmental conditions. The distinct metabolic profiles varied considerably among germplasm resources and were not strongly associated with geographical origin, suggesting that genetic background plays an important role in determining nutritional quality. Organic acids, flavonoids, and triterpenoids were among the major metabolite classes associated with differences in metabolic profiles among germplasms, and several germplasm resources showed distinctive profiles characterized by relatively high abundance of multiple functional metabolite classes. These findings provide new insights into the metabolic diversity of dried jujube and the contribution of germplasm-associated variation to nutritional quality under common-garden conditions.

## Data Availability

The original contributions presented in the study are included in the article/**Supplementary Material**. Further inquiries can be directed to the corresponding author.
