# Supplementary material for: Common-garden metabolomics reveals germplasm-associated metabolic diversity and candidate resources for functional food development in dried jujube (Ziziphus jujuba Mill.)
Source: Front Plant Sci. 2026 Oct 14;17:1980416. doi: 10.3389/fpls.2026.1980416 (PMC13713513; doi:10.3389/fpls.2026.1980416)
Supplement: Supplementary file 1 [file DataSheet1.pdf]

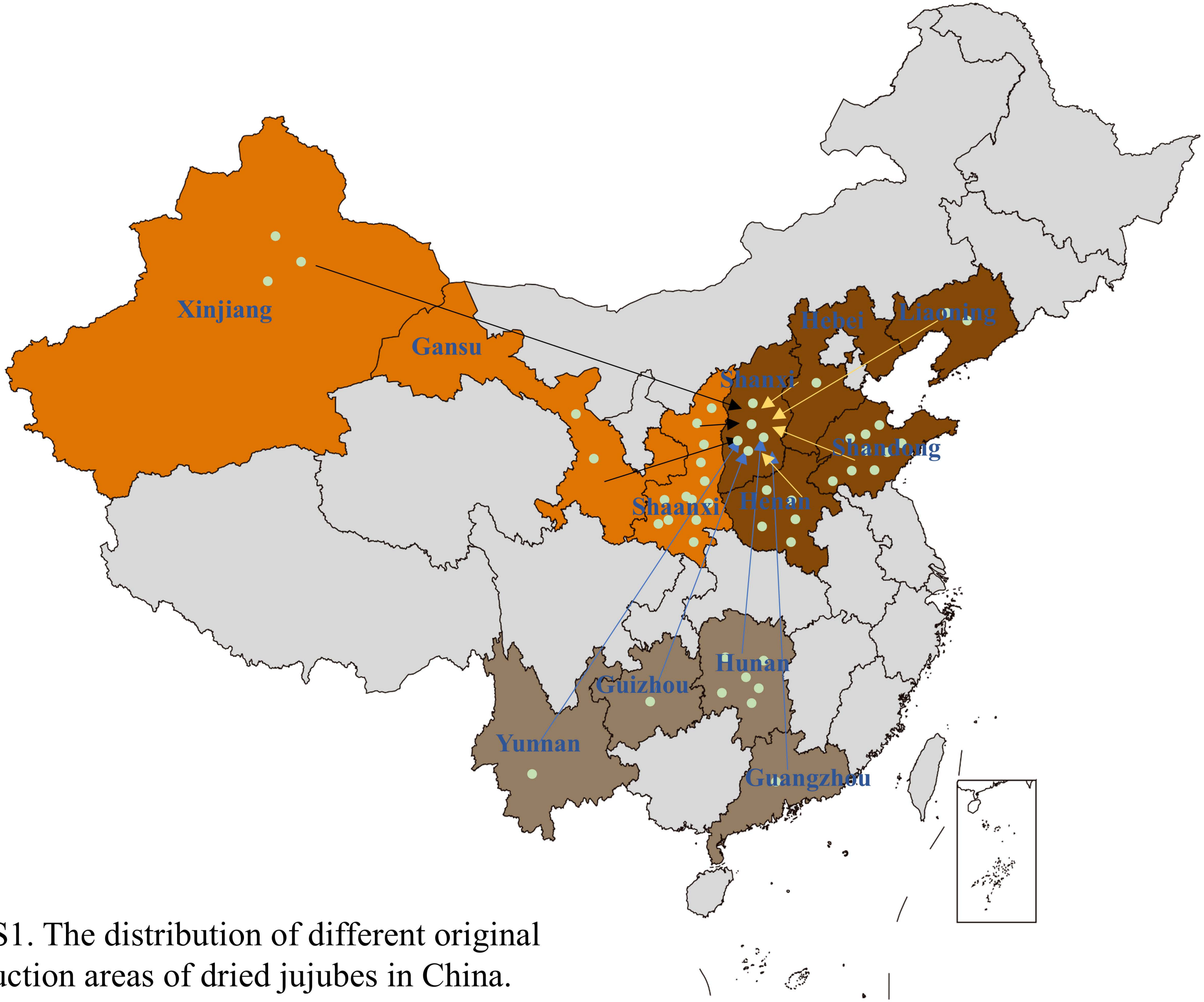

Fig. S1. The distribution of different original production areas of dried jujubes in China.

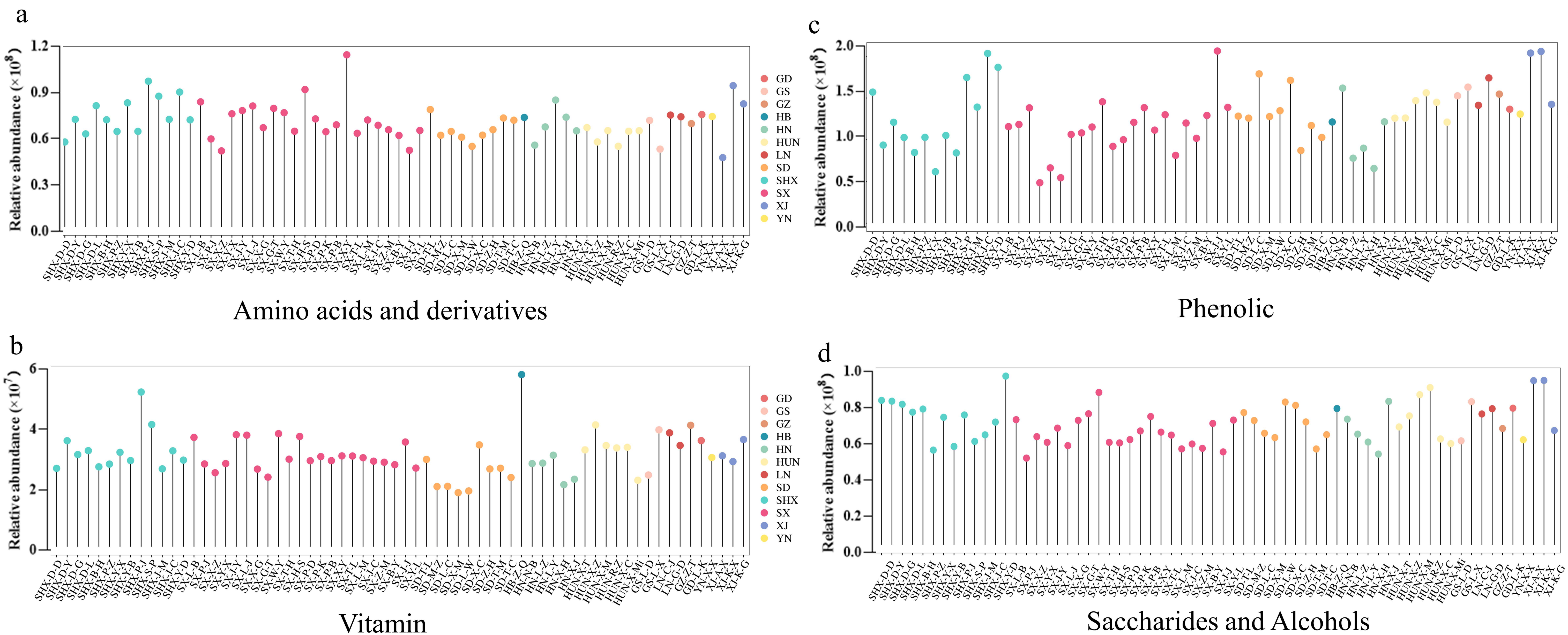

Fig. S2. Analysis of key metabolite contents in different types of dried jujubes. (a) Amino acids and derivatives; (b) Vitamin; (c) Phenolic; (d) Saccharides and alcohols.
